# Analysis of microsatellites in the vulnerable orchid *Gastrodia flavilabella*: the development of microsatellite markers, and cross-species amplification in *Gastrodia*

**DOI:** 10.1186/s40529-014-0072-4

**Published:** 2014-10-09

**Authors:** Chi-Chu Tsai, Pei-Yin Wu, Chia-Chi Kuo, Min-Chun Huang, Sheng-Kun Yu, Tsai-Wen Hsu, Tzen-Yuh Chiang, Yu-Chung Chiang

**Affiliations:** 1 Crop Improvement Division, Kaohsiung District Agricultural Research and Extension Station, Pingtung, 908 Taiwan; 2 Department of Life Science, National Cheng Kung University, Tainan, 701 Taiwan; 3 Department of Nursing, Meiho University, Pingtung, 912 Taiwan,; 4 Department of Biological Sciences, National Sun Yat-sen University, Kaohsiung, 804 Taiwan; 5 Taiwan Society of Plant Systematics, Kaohsiung, 804 Taiwan; 6 Endemic Species Research Institute, Nantou, 552 Taiwan

**Keywords:** Gastrodia, Conservation, Microsatellites, Mycoheterotrophic orchid, Population genetics, Simple sequence repeat markers

## Abstract

**Background:**

*Gastrodia flabilabella* is a mycoheterotrophic orchid that obtains carbohydrates and nutrients from its symbiotic mycorrhizal fungi. The species is an endemic and vulnerable species enlisted in the “A Preliminary Red List of Taiwanese Vascular Plants” according to the IUCN Red List Categories and Criteria Version 3.1. *G. flabilabella* dwells the underground of broadleaf and coniferous forest with richness litter. Based on herbarium records, this species is distributed in central Taiwan. Twenty eight microsatellite loci were developed in *G. flabilabella* and were tested for cross-species amplification in additional taxa of *G. confusoides*, *G. elata*, and *G. javanica*. We estimated the genetic variation that is valuable for conservation management and the development of the molecular identification system for *G. elata*, a traditional Chinese medicine herb.

**Results:**

Microsatellite primer sets were developed from *G. flabilabella* using the modified AFLP and magnetic bead enrichment method. In total, 257 microsatellite loci were obtained from a magnetic bead enrichment SSR library. Of the 28 microsatellite loci, 16 were polymorphic, in which the number of alleles ranged from 2 to 15, with the observed heterozygosity ranging from 0.02 to 1.00. In total, 15, 13, and 7 of the loci were found to be interspecifically amplifiable to *G. confusoides*, *G. elata*, and *G. javanica*, respectively.

**Conclusions:**

Amplifiable and transferable microsatellite loci are potentially useful for future studies in investigating intraspecific genetic variation, reconstructing phylogeographic patterns among closely related species, and establishing the standard operating system of molecular identification in *Gastrodia*.

## Background

*Gastrodia* is the largest achlorophyllous and mycoheterotrophic genus in the Orchidaceae with 50 to 60 species in the world. Recent studies recognized 19 species, including 13 endemic species distributed in Taiwan (Hsu [2008]; Leou [2000]; Hsu and Kuo [2010]; Chung and Hsu [2006]). Species diversity in Taiwan Island is one of the hot spots of *Gastrodia* in the world. *Gastrodia elata* Blume is an important Chinese medicine that provides supplement to protect neuron and cardiovascular systems (Baek et al. [1999]). Ecologically, *Gastrodia* species are saprophyte (Leou [2000]), growing underground of forest or bamboo grove with richness litter and obtaining carbohydrates and nutrients from its symbiotic mycorrhizal fungi, including *Armillaria mellea* and other microbial species (Cha and Igarashi [1995]). Due to such a unique growth form, *Gastrodia* species are difficult to find except the flowering and fruiting seasons, generally 2 to 4 weeks after budding. Most *Gastrodia* species are vulnerable to the human destruction. As a result, 7 species recognized as threatened species, including one as critically endangered, three as endangered, and another three as vulnerable, are evaluated by the IUCN Red List Categories and Criteria Version 3.1 (IUCN [2012]) and listed in the “A Preliminary Red List of Taiwanese Vascular Plants” (Wang et al. [2012a]).

*Gastrodia flavilabella* S.S. Ying is an endemic and vulnerable species with only few populations distributed at the edges of conifer plantation or natural broadleaf forests restricted to the central mountainous regions from 1,000 to 1,300 meters altitude (Leou [2000]). This taxon is characterized by tuberous horizontal rhizomes ca. 4 to 10 cm in length and 0.6–1.6 cm in width bearing many coral-like buds (Leou [2000]). Unique life form and habitat preference lead his species to be rare and vulnerable. However, no data for the genetic diversity in this species or genus are available, which is critical for evaluating the population dynamics and conservation genetics for conservation management.

Microsatellite genotyping is the most popular molecular tool for evaluating the structure and genetic diversity of populations because of its high genetic variability (cf. Ho et al. [2014]). With co-dominant inheritance, the information of microsatellite genotyping can estimate the effective population sizes in ancestral and present populations (Ge et al. [2014]), Hardy-Weinberg Equilibrium (Ge et al. [2012]), and levels of introgression (Liao et al. [2012]). In addition, microsatellite genotyping technology was extended to molecular identification system for paternity testing and cultivar identification (Tsai et al. [2013]).

In this study, we constructed a microsatellite enriched library and developed microsatellite loci for future estimating the population genetic diversity based on microsatellite genotyping. The application of the microsatellite primers developed in this study was tested in other taxa of *Gastrodia*, specifically three taxa for polymorphism test and 13 species for transferability test.

## Methods

### Sampling and DNA extractions

Twenty individuals from each of four taxa in *Gastrodia*, including *G. flavilabella* from Nantou, *G. elata* from China, *G. javanica* (Blume) Lindl. from Lanyu Islet, and *G. confusoides* T. C. Hsu, S. W. Chung & C. M. Kuo from Taichung (Table 1) were sampled for polymorphism test. One individual of *G. flavilabella* was used to construct a microsatellite enriched library and to develop microsatellite loci. To test the transferability of these newly designed microsatellite primers, two individuals of other 13 native taxa listed in Table 1, specifically 8 endemic species, were sampled from the field. The sample location, sample size, and deposited herbarium for the voucher specimens are listed in Table 1. To avoid the contamination from the symbiotic mycorrhizal fungi, we collected the flower buds or seed pods for extracting total genomic DNA. Total DNA was extracted from silica-dried plant materials using the Plant Genomic DNA Extraction Kit (RBC Bioscience, Taipei, Taiwan).

**Table 1:** **Sample location for each species of the** ***Gastrodia***

Species	Location	Species code	Sample size	Latitude	Longitude	Herbarium
*Gastrodia flavilabella*	Nantou, Taiwan	Gfl	20	N 23°39′43″	E 120°47′41″	TAIE
*Gastrodia elata*	Yunan, China	Gel	20	N 27°46′07″	E 104°15′39″	TAIE
*Gastrodia javanica*	Lanyu, Taiwan	Gja	20	N 22°00′53″	E 121°34′17″	TAIE
*Gastrodia confusoides*	Taichung, Taiwan	Gco	20	N 24°14′21″	E 120°54′81″	TAIE
*Gastrodia albida*	Taipei, Taiwan	Gal	2	N 24°50′36″	E 121°33′28″	TAIE
*Gastrodia appendiculata*	Nantou, Taiwan	Gap	2	N 23°41′17″	E 120°47′26″	TAIE
*Gastrodia autumnalis*	Taoyuan, Taiwan	Gau	2	N 24°47′34″	E 121°26′08″	TAIE
*Gastrodia clausa*	Taipei, Taiwan	Gcl	2	N 25°04′57″	E 121°37′33″	TAIE
*Gastrodia fontinalis*	Taipei, Taiwan	Gfo	2	N 24°51′27″	E 121°32′19″	TAIE
*Gastrodia gracilis*	Chaiyi, Taiwan	Ggr	2	N 23°29′28″	E 120°43′42″	TAIE
*Gastrodia leoui*	Chaiyi, Taiwan	Gle	2	N 23°29′28″	E 120°43′42″	TAIE
*Gastrodia nantoensis*	Nantou, Taiwan	Gna	2	N 23°41′17″	E 120°47′27″	TAIE
*Gastrodia nipponica*	Taipei, Taiwan	Gni	2	N 24°51′05″	E 121°32′11″	TAIE
*Gastrodia pubilabiata*	Nantou, Taiwan	Gpu	2	N 23°40′23″	E 120°47′54″	TAIE
*Gastrodia shimizuana*	Pingtung, Taiwan	Gsh	2	N 22°12′12″	E 120°47′16″	TAIE
*Gastrodia theana*	Nantou, Taiwan	Gth	2	N 23°51′57″	E 120°55′42″	TAIE
*Gastrodia uraiensis*	Taipei, Taiwan	Gur	2	N 24°50′41″	E 121°33′34″	TAIE

Note: TAIE = the herbarium of the Taiwan Endemic Species Research Institute.

Sample size, location, coordinates, and voucher specimens are indicated.

### Isolation of microsatellite DNA loci and identification

In order to develop the molecular markers for evaluating the genetic variation of populations and testing transferability in *Gastrodia* species, we selected one individual of *G. flabilabella* to build (AG)n, (AC)n, (TTG)n, (TCC)n, (ACG), (CCA)n, (AACT)n, and (AGAT)n enrich DNA library. Microsatellite loci were isolated following the magnetic bead enrichment method (Liao et al. [2009]; Hsu et al. [2013]), modified from the method proposed by Zane et al. ([2002]) based on AFLP, magnetic bead enrichment, and TA cloning protocol. Genomic DNA of *G. flabilabella* was digested using the restriction enzyme *Mse* I (Promega, Madison, Wisconsin, USA) and DNA fragments from 400 to 1000 bps were isolated from agarose gels using the HiYield^TM^ Gel PCR DNA Fragments Extraction Kit (RBC Bioscience). The purified partial genomic library was ligated to adaptors (complementary oligo A: 5′-TACTCAGGACTCAT-3′ and 5′ phosphorylated oligo B: 5′-GACGATGAGTCCTGAG-3′). The partial genomic library was enriched using 15 cycles of prehybridization polymerase chain reaction (PCR) using adaptor specific primers (5′-GATGAGTCCTGAGTAAN-3′, hereafter referred to as *Mse* I-N). The enriched partial genomic library was denatured and hybridized to eight different biotinylated probes [Biotin-(AG)_15_, Biotin-(AC)_15_, Biotin-(TTG)_10_, Biotin-(TCC)_10_, Biotin-(ACG)_10_, Biotin-(CCA)_10_, Biotin-(AACT)_8_, and Biotin-(AGAT)_8_] at 68°C for 1 hour for enrichment. The DNA fragments hybridized to probes was incubated and captured using Streptavidin MagneSphere Paramagnetic Particles (Promega) at 42°C for 2 hours. The microsatellite enriched DNA fragments were eluted with high- and low-salt solutions and used as template DNAs for 25 cycles of PCR amplification. The microsatellite enriched DNA fragments were then used as templates for 25 cycles of PCR amplification using *Mse* I-N. The PCR products were purified using the HiYield^TM^ Gel PCR DNA Fragments Extraction Kit (RBC Bioscience) and then cloned directly into the *p* GEM^®^-T Easy Vector System (Promega). Plasmids containing the PCR product were isolated using an alkaline lysis protocol (Birnboim and Doly [1979]), screened using PCR with primer pairs: (AG)_10_ or (AC)_10_/SP6 or T7), and purified with a PureYield^TM^ Plasmid Miniprep System (Promega). The selected plasmids were subsequently sequenced in both directions using an ABI BigDye3.1 Terminator Cycle Sequencing Kit (Applied Biosystems, USA) with the ABI PRISM^®^ 3700 DNA Automated Sequencer. Sequences enclosing tandem repeat sequences were recognized using Tandem Repeats Finder version 4.07b (Benson [1999]) by general setting on 2, 3, and 5 of match, mismatch, and indel for alignment parameters and 20 for minimum alignment score to report repeat. The pair of specific primers for each microsatellite locus detected by Tandem Repeats Finder was designed using FastPCR software version 6.4.18 (Kalendar et al. [2011]) based on the setting of parameters at a PCR product size ranging from 100 to 400 bp, an optimum annealing temperature of 55°C, and a GC content ranging from 35% to 70%.

### DNA amplification and genotyping

To optimize PCR at various annealing temperatures, we evaluated each primer pair using a gradient PCR procedure. All primer pairs were tested for PCR amplification on DNA extracted from each species, i.e., two individuals of each 17 taxa. The protocol was executed at 94°C for 5 min, followed by 30 cycles of 94°C for 30 s, 48–65°C for 30 s, 72°C for 30 s, and a final extension of 72°C for 10 minutes with the LabnetMultiGene 96-well Gradient Thermal Cycler (Labnet, Edison, NJ, USA). PCR products were checked by 10% PAGE electrophoresis to separate the target DNA bands and which were following confirmed based on cloning and sequencing. These SSR primer pairs with confirmed target DNA bands were chosen for polymorphism evaluation.

To investigate genetic polymorphisms, 20 individuals from each of four taxa were selected (Table 1). PCR reaction cocktail contained 20 ng template DNA, 0.2 μM each of forward and reverse primers, 2 μL 10 × PCR reaction buffer, 2 mM dNTP mix, 2 mM MgCl_2_, 0.5 U *Taq* DNA polymerase (Promega), plus adding sterile water to total volume to 20 μL. PCR amplifications were executed by a Labnet MultiGene 96-well Gradient Thermal Cycler (Labnet). The PCR protocol was piloted at 94°C for 5 min, followed by 30 cycles of 94°C for 30 s, at the optimal annealing temperature (*Ta*) for 30 s, 72°C for 30 s, and a final extension of 72°C for 10 minutes (Chiang et al. [2012]). PCR products were separated by electrophoresis on a 10% polyacrylamide gel (acrylamide: bisacrylamide 29: 1, 80 V for 14–16 hours) and determined the allele size by a 25 or 50 bp DNA Step Ladder (Promega). The bands of amplicons were then imaged under UV light using the Flo Gel FGIS-3 fluorescent gel image system (Top BIO Co., Taipei, Taiwan), and the sizes of bands were estimated using Quantity One software version 4.62 (Bio-Rad Laboratories, Hercules, California, USA).

### Genetic variation analysis

Several genetic variation parameters were calculated using GenAlEx version 6.5 (Peakall and Smouse [2012]), including the number of alleles (*Na*), the number of effective alleles (*Ne*), the observed and expected heterozygosity (*Ho* and *He*), Shannon’s information index (*H*), fixation index (*F*_*IS*_). Hardy–Weinberg equilibrium (*H*_*WE*_) was evaluated using Arlequin software version 3.5.1.2 (Excoffier and Lischer [2010]).

## Results and discussion

### Enrichment microsatellite library and sequencing results

We sequenced 1047 positive plasmids from eight microsatellite enrich libraries and confirmed 257 microsatellite loci from SSR enrich library (Table 2). Among the derived repeats of microsatellite loci, the di-, tri-, tetra-, penta-, and hexanucleotide motif was existed in 106 (41.25%), 120 (46.69%), 9 (3.05%), 11 (4.28%) and 9 (1.67%) loci, respectively (Table 2). Di- (41.25%) and trinucleotide repeats (46.69%) comprised the largest group of repeat motifs and accounted for more than four-fifths of the total SSR content, while the rest amounted to less than 12.06%. Generally, di- and trinucleotide repeats overstepped other types of repeats in all the species and mostly contributed to the major fraction of SSRs (Wei et al. [2014]). Among the repeat motifs within *G. flavilabella*, di- and trinucleotide repeats were the commonest motifs, representing for 87.94%, similar to *Sesamum indicum* (Wei et al. [2014]), *Arabidopsis thaliana*, *Sorghum bicolor* (Sonah et al. [2011]), and *Brassica napus* (Cheng et al. [2009]).

**Table 2:** **Summary of different SSR repeat motif types related to variation of repeat unit numbers in 257** ***Gastrodia flavilabella*** **SSR loci selected by the length of repeat motif more than 20 bps**

No. of repeat units	Di-	Tri-	Tetra-	Penta-	Hexa-	Mix	Total
4	1	13	8	1	0	0	23
5	0	5	0	1	0	0	6
6	2	4	0	0	1	0	7
7	5	4	0	0	0	0	9
8	4	3	0	0	0	0	7
9	3	4	0	0	0	0	7
10	6	4	0	0	2	0	12
11	2	4	0	0	0	0	6
12	3	3	0	0	2	0	8
≥13	80	76	1	9	4	2	172
Total	106	120	9	11	9	2	257

### Development of microsatellite markers

Totally, we designed 144 microsatellite primer pairs based on the flanking sequences from 257 microsatellite loci. All primer pairs were screened using a gradient PCR protocol with a Labnet MultiGene^TM^ 96-well Gradient Thermal Cycler (Labnet) to find the best annealing temperature. Finally, 28 primer pairs showed desired DNA bands and were selected for future diversity evaluation. The characteristics of 28 microsatellite loci are listed in Table 3. Of the 28 loci, 26 are complete microsatellite loci, including 13 carrying a dinucleotide motif, 11 with a trinucleotide motif, 1 with a pentanucleotide motif, and 1 with a hexanucleotide motif, and 2 remaining loci are carried a compound motif. The sequences of 28 loci reported in this paper are available from GenBank (accession numbers: LK934509–LK934536) (Table 3).

**Table 3:** **Summary of general information for the 28 microsatellite loci isolated from** ***Gastrodia flavilabella***

Locus	Repeat motif	Primer sequence (5′-3′)	Allele size (bps)	***Ta***(°C)				Genbank accession no.
				Gfl	Gel	Gja	Gco	
CT3-32	(GGA)_9_	F: TAACGGGGAATGGGGAGGCG	137–146	52	-	54	-	LK934509
		R: TTGCGATCCCTCCCCTGTAC						
CT6-4	(GA)_29_	F: CAAGAATAGGTGCCAACCTC	110–151	55	-	-	-	LK934510
		R: GTGAGTTACTAGCGTGCGGC						
CT6-35	(TG)_84_	F: GTCTGTTCCATTTGATATTG	250–252	55	-	-	50	LK934511
		R: GCAGTAATGACCTTTGTAGT						
CT6-65	(TGT)_36_	F: CACCGAGCTTTTTGTCAATG	247–262	55	52	-	51	LK934512
		R: GCAATAACAATAGTAGCAGC						
CT6-90	(TTG)_7_	F: CAACCAAGACAAGACTCATG	132	55	55	52	55	LK934513
		R: ACATTCTTCCCTGGATGTTC						
CT6-99	(CAA)_7_	F: GGCATTATCCTGTTATACTC	138	55	50	-	55	LK934514
		R: GGGCTTTCATTTGATCATGC						
CT6-120	(CACAG)_38_	F: TAGCAGCCATAAGTAAAGCC	316	55	-	-	-	LK934515
		R: GTCGAGGATCAAATGAATTG						
CT6-142	(AAC)_7_	F: GTCATGCACATTCTTCCCTG	128–131	55	55	-	55	LK934516
		R: AGACTCATGTTGTTGATCCC						
CT-ACT-74	(AG)_29_	F: GAGGTCCAATCTAAGATTTC	122–156	54	-	-	-	LK934517
		R: CATGATATAATTCTCACCCC						
CT-ACT-88	(TGA)_9_	F: TAGTGGATTTGGAGTTTGAG	101	54	-	-	51	LK934518
		R: CTCATCTTTGATACCTCTTC						
CT-ACT-136	(CT)_12_	F: ATTTAGGGTCATCGAGCACC	140–142	54	55	55	54	LK934519
		R: TCGGCAAGGTGTCAAGACTC						
CT-AG-35	(GA)_12_	F: TCTTCCCGCACCTCTTCAAC	133–137	52	55	55	55	LK934520
		R: TTCAGAAGCATGGCACTGGG						
CT-AG-45	(CTT)_12_	F: CAGAAGCCAACATATCCATC	115–121	50	54	-	52	LK934521
		R: TCTGAAATTTAGTGTAGCGG						
CT-AG-55	(TGCCTC)_5_	F: GTGGGGAGATTACTATTACG	108–110	50	50	-	55	LK934522
		R: AAGGAAAGGCGTAAGGATAG						
CT-AG-85	(TG)_9_ (AG)_28_	F: CCCATATGTCCTTGGTCATC	208–248	54	-	-	-	LK934523
		R: GCTTACAACTTTCTCCCTTC						
CT-AG-88	(AG)_15_	F: ACAACCTACACTGTCTAAAG	152	55	54	-	55	LK934524
		R: CTTTTTTTGTGTGGTCACCG						
CT-AG-114	(TG)_13_	F: AGTGATATGATAACACCCTC	104	50	-	-	-	LK934525
		R: TAGATCTCTAGCTTCAACTC						
CT-AG-127	(TC)_9_	F: AAGCTTCGCTGCCCTCTTCG	117–123	54	-	-	-	LK934526
		R: TTGGTTTCGGGCCAGAGCTG						
CT-AG-140	(AG)_15_	F: AGTCCTGCCTTCAAGCCTTG	120–126	54	55	55	55	LK934527
		R: GAAGGATTCAAGCATGGGAG						
CT-AG-144	(AG)_18_	F: GGCGATGTCAATTCAACAAG	113–115	52	55	55	55	LK934528
		R: TAACGATAGCTGCCTTCCAC						
CT-AG-145	(TC)_14_ (ACTC)_3_	F: ATCTTCGTACATCTAACCCG	140	54	-	-	55	LK934529
		R: AATGAGCTCGTTGCAGCTTC						
CT-AG-157	(TG)_14_	F: TGCAGTAATAGCATTTGCAG	120	56	55	-	55	LK934530
		R: AGGCTGCCACTGTACTTTTC						
CT-AGAT-19	(TC)_19_	F: TACATTGATTAGGATGCCTC	169	55	50	-	50	LK934531
		R: ACATTTGTGCCTCCTCCAAC						
CT-AGAT-26	(TG)_88_	F: GAATGATGCTATGTGTGCTG	295	55	-	-	-	LK934532
		R: TGCAGTAATAGCATTTGCAG						
CT-AGAT-131	(CCA)_7_	F: TTCAATCGCTAGTAGCTCTG	139	55	-	-	50	LK934533
		R: GTTGACATTTAGTGGAGAGG						
CT-CCA-71	(TGG)_14_	F: ACATGAGTAGGAGCATCCTC	150–156	50	-	-	50	LK934534
		R: TTTCTCTTCCCCACAGCTGC						
CT-CCA-108	(CCA)_127_	F: CATGGTGGGACATAAAACTG	489–516	47	-	-	-	LK934535
		R: GTGGTTGTAGTCATCACTCC						
CT-CCA-137	(CCA)_6_	F: AATCTCAGAGCCTTTCCCAG	150	55	-	-	55	LK934536
		R: TTGGAGGTTGCTTGTAGAGC						

Note: F = the forward primer; R = the reverse primer; *T* a = optimized annealing temperature.

### Genotyping and population genetics analysis

To inspect the level of genetic polymorphism at each locus, 20 individuals were collected in the field from the remaining wild population of *G. flabilabella* (Table 1). All the 28 new microsatellite loci identified in *G. flabilabella* were successfully amplified. Of the 28 loci, 12 microsatellite loci were monomorphic and 16 were polymorphic (Table 4). Genetic variation indices for 16 polymorphic loci, including the number of alleles (*Na*), the number of effective alleles (*Ne*), the observed and expected heterozygosity (*Ho* and *He*), Shannon’s information index (*H*) and fixation index (*F*_*IS*_), were estimated. *Ne* represents here an estimate of the number of equally frequent alleles in a model population following the formula of *Ne* = 1/ (1- *He*). As shown in Table 4, *Na* ranged from 2 to 15, *Ne* varied from 1.08 to 8.85, *Ho* ranged from 0 to 1.00 and mean was 0.163, and *He* varied from 0.08 to 0.89 and mean was 0.444. The Shannon’s information index (*H*) and fixation index (*F*_*IS*_) ranged from 0.17 to 2.41 and from -1.00 to 1.00, and the mean was 0.882 and 0.697, respectively. Significant deviations from Hardy–Weinberg equilibrium (*H*_*WE*_) were detected at all loci (Table 4).

**Table 4:** **Genetic diversity characteristics of the 28 microsatellite loci tested on four** ***Gastrodia*** **taxa**

	***Gastrodia flavilabella***						***Gastrodia elata***						***Gastrodia javanica***						***Gastrodia confusoides***					
Locus	***Na***	***Ne***	***Ho***	***He***	***H***	***F***_***IS***_	***Na***	***Ne***	***Ho***	***He***	***H***	***F***_***IS***_	***Na***	***Ne***	***Ho***	***He***	***H***	***F***_***IS***_	***Na***	***Ne***	***Ho***	***He***	***H***	***F***_***IS***_
CT3-32	4	1.23	0.00	0.19*	0.43	─	─	─	─	─	─	─	1	1.00	─	─	─	─	─	─	─	─	─	─
CT6-4	15	8.85	0.10	0.89*	2.41	0.887	─	─	─	─	─	─	─	─	─	─	─	─	─	─	─	─	─	─
CT6-35	2	1.08	0.00	0.08*	0.17	1.000	─	─	─	─	─	─	─	─	─	─	─	─	─	─	─	─	─	─
CT6- 65	5	2.94	0.32	0.66*	1.23	0.515	1	1.00	─	─	─	─	─	─	─	─	─	─	2	1.06	0.06	0.06	0.13	-0.030
CT6-90	1	1.00	─	─	─	─	1	1.00	─	─	─	─	1	1.00	─	─	─	─	1	1.00	─	─	─	─
CT6-99	1	1.00	─	─	─	─	1	1.00	─	─	─	─	─	─	─	─	─	─	1	1.00	─	─	─	─
CT6-120	1	1.00	─	─	─	─	─	─	─	─	─	─	─	─	─	─	─	─	─	─	─	─	─	─
CT6-142	2	1.04	0.00	0.04*	0.10	1.000	1	1.00	─	─	─	─	─	─	─	─	─	─	1	1.00	─	─	─	─
CT-ACT-74	10	4.03	0.16	0.75*	1.80	0.783	─	─	─	─	─	─	─	─	─	─	─	─	─	─	─	─	─	─
CT-ACT-88	1	1.00	─	─	─	─	─	─	─	─	─	─	─	─	─	─	─	─	─	─	─	─	─	─
CT-ACT-136	2	2.00	1.00	0.50*	0.69	-1.000	1	1.00	─	─	─	─	2	2.00	1.00	0.50*	0.69	-1.000	1	1.00	─	─	─	─
CT-AG-35	3	2.56	0.00	0.61*	1.00	1.000	7	4.37	0.05	0.77*	1.64	0.935	2	1.11	0.11	0.10	0.21	-0.056	2	1.11	0.00	0.10*	0.20	1.000
CT-AG-45	3	1.09	0.00	0.08*	0.20	1.000	1	1.00	─	─	─	─	─	─	─	─	─	─	1	1.00	─	─	─	─
CT-AG-55	2	1.95	0.00	0.49*	0.68	1.000	1	1.00	─	─	─	─	─	─	─	─	─	─	2	2.00	1.00	0.50*	0.69	-1.000
CT-AG-85	8	3.97	0.02	0.75*	1.62	0.972	5	3.86	1.00	0.74*	1.43	-0.349	─	─	─	─	─	─	─	─	─	─	─	─
CT-AG-88	1	1.00	─	─	─	─	─	─	─	─	─	─	─	─	─	─	─	─	7	4.35	1.00	0.77*	1.64	-0.299
CT-AG-114	1	1.00	─	─	─	─	─	─	─	─	─	─	─	─	─	─	─	─	─	─	─	─	─	─
CT-AG-127	3	1.14	0.00	0.12*	0.28	1.000	─	─	─	─	─	─	─	─	─	─	─	─	─	─	─	─	─	─
CT-AG-140	4	2.34	0.00	0.57*	0.97	1.000	4	1.49	0.00	0.33*	0.69	1.000	1	1.00	─	─	─	─	1	1.00	─	─	─	─
CT-AG-144	2	1.17	0.00	0.15*	0.28	1.000	1	1.00	─	─	─	─	1	1.00	─	─	─	─	1	1.00	─	─	─	─
CT-AG-145	1	1.00	─	─	─	─	─	─	─	─	─	─	─	─	─	─	─	─	1	1.00	─	─	─	─
CT-AG-157	1	1.00	─	─	─	─	1	1.00	─	─	─	─	1	1.00	─	─	─	─	1	1.00	─	─	─	─
CT-AGAT-19	1	1.00	─	─	─	─	2	2.00	1.00	0.50*	0.69	-1.000	─	─	─	─	─	─	2	2.00	1.00	0.50*	0.69	-1.000
CT-AGAT-26	1	1.00	─	─	─	─	─	─	─	─	─	─	─	─	─	─	─	─	─	─	─	─	─	─
CT-AGAT-131	1	1.00	─	─	─	─	─	─	─	─	─	─	─	─	─	─	─	─	1	1.00	─	─	─	─
CT-CCA-71	2	2.00	1.00	0.50*	0.69	-1.000	─	─	─	─	─	─	─	─	─	─	─	─	1	1.00	─	─	─	─
CT-CCA-108	7	3.71	0.00	0.73*	1.57	1.000	─	─	─	─	─	─	─	─	─	─	─	─	─	─	─	─	─	─
CT-CCA-137	1	1.00	─	─	─	─	─	─	─	─	─	─	─	─	─	─	─	─	1	1.00	─	─	─	─
Mean	3.071	1.896	0.163	0.444	0.882	0.697	2.077	1.480	0.513	0.585	1.113	0.147	1.286	1.159	0.555	0.300	0.450	-0.528	1.588	1.324	0.612	0.386	0.67	-0.266

The number of different alleles (*Na*), number of effective alleles (*Ne*), observed heterozygosity (*H*_*O*_), expected heterozygosity (*He*), Shannon’s information index (*H*), and fixation index (*F*_*IS*_) are reported.

*Significant deviation from Hardy-Weinberg equilibrium: P < 0.05.

To test the transferability and genetic diversity, 20 individuals from each of three taxa, including *G. elata*, *G. javanica*, and *G. confusoides*, were tested. Of the 28 loci, 13, 7, and 17 markers worked in *G. elata*, *G. javanica*, and *G. confusoides*, respectively. Of the 13, 7, and 17 microsatellite loci, 9, 5, and 12 were monomorphic and 4, 2, and 5 were polymorphic (Table 4). In addition, three loci, including CT6-90, CT6-99, and CT-AG-157, are monomorphic within each of four species, but polymorphic between species. As shown in Table 4, the ranges for the *Na*, *Ne*, *Ho* and *He* were varied from 1 to 7, 1.00 to 4.37, 0.00 to 1.00, and 0.33 to 0.77 in *G. elata*, 1 to 2, 1.00 to 2.00, 0.11 to 1.00, and 0.10 to 0.50 in *G. javanica*, and 1 to 7, 1.00 to 4.35, 0.00 to 1.00, and 0.06 to0.77 in *G. confusoides*. The Shannon’s information index (*H*) and fixation index (*F*_*IS*_) ranged from 0.69 to 1.64 and from -1.00 to 1.00, and the mean was 1.113 and 0.147 in *G. elata*, from 0.21 to 0.69 and from -1.00 to -0.056, and the mean was 0.450 and -0.528 in *G. javanica*, and from 0.13 to 1.64 and from -1.00 to -0.056, and the mean was 0.670 and -0.266 in *G. javanica*. Significant deviations from Hardy–Weinberg equilibrium (*H*_*WE*_) were detected at 4 of 4, 1 of 2, and 4 of 5 polymorphic loci (Table 4).

For orchids, only few researches were used simple sequence repeats to evaluate the genetic diversity. The genetic diversity, including the means of the observed (*Ho*) and expected heterozygosity (*He*) (Table 4), of *G. flabilabella* was low compared with that of other Orchidaceae species, such as *Dendrobium huoshanense* (0.512 and 0.569) (Wang et al. [2012b]), *Dendrobium officinale* (0.720 and 0.740) (Xie et al. [2010]), *Dendrobium officinale* (0.514 for *Ho*) (Lu et al. [2012]), and *Dendrobium nobile* (0.350 and 0.608) (Lu et al. [2014]). Unfortunately, no data for any *Gastrodia* taxa or mycoheterotrophic orchids are available for the comparison of genetic variability. However, the low observed and expected heterozygosity values implied that rare and mycoheterotrophic taxa tend to possess low levels of genetic diversity due to stochastic losses of genetic polymorphisms resulting from genetic drift (cf. Ge et al. [2014]). In addition, significant deviations from Hardy–Weinberg equilibrium (*H*_*WE*_) were detected at all loci in the remained population, and these deviations were credited to the heterozygote deficiency likely due to the unique interactions between orchids and pollinators (Boberg et al. [2014]). Besides, the habitat preferences (Mallet et al. [2014]) strengthened the isolation among populations.

### Test the transferability

To test the transferability of these microsatellite loci, we tested the primers in 13 other *Gastrodia* taxa (Table 1). Two individuals of each taxon were used in the evaluation of cross-amplification. Of the 28 loci,11 to 17 loci were transferable to each of the 13 taxa of *Gastrodia* (Table 5), and the annealing temperatures are listed on Table 3. Three loci, including CT-ACT-136, CT-AG-88, and CT-AG-144, were transferable, and four loci, including CT6-4, CT6-35, CT-AG-85, and CT-AG-114, did not work in all taxa (Table 5). In addition, 13 of 28 loci successfully amplifying more than 10 taxa will be useful across species. Nonetheless, population genetics, phylogeographic patterns, and process of speciation among the *Gastrodia* taxa remain unclear. The primer set of these 13 microsatellite markers with high transferability represents a useful tool of genetic markers for interspecific researches.

**Table 5:** **Result of cross-species transferability in 13** ***Gastrodia*** **taxa using the 28 microsatellite primers developed from** ***Gastrodia flavilabella***

	Gal	Gap	Gau	Gcl	Gfo	Ggr	Gle	Gna	Gni	Gpu	Gsh	Gth	Gur	Total
Locus	(N = 2)	(N = 2)	(N = 2)	(N = 2)	(N = 2)	(N = 2)	(N = 2)	(N = 2)	(N = 2)	(N = 2)	(N = 2)	(N = 2)	(N = 2)	Species
CT3-32	─	─	─	─	─	─	─	─	1	─	1	1	─	3
CT6-4	─	─	─	─	─	─	─	─	─	─	─	─	─	0
CT6-35	─	─	─	─	─	─	─	─	─	─	─	─	─	0
CT6- 65	1	1	1	1	─	1	─	1	1	1	1	1	─	10
CT6-90	─	1	1	1	1	─	1	1	1	1	─	1	1	10
CT6-99	─	1	1	1	1	1	1	1	1	1	1	1	1	12
CT6-120	─	─	─	─	─	─	─	─	1	─	─	─	─	1
CT6-142	1	1	1	1	1	1	─	1	1	1	1	1	1	12
CT-ACT-74	─	─	─	─	1	1	─	─	─	1	1	─	─	4
CT-ACT-88	1	─	1	─	─	1	─	─	─	─	1	1	─	5
CT-ACT-136	1	1	1	1	1	1	1	1	1	1	1	1	1	13
CT-AG-35	1	1	1	1	1	─	2	1	1	1	1	─	1	11
CT-AG-45	1	─	1	1	─	─	1	1	1	1	─	1	─	8
CT-AG-55	1	─	1	1	1	1	1	1	1	1	1	1	1	12
CT-AG-85	─	─	─	─	─	─	─	─	─	─	─	─	─	0
CT-AG-88	1	1	1	2	1	1	1	1	1	1	1	1	1	13
CT-AG-114	─	─	─	─	─	─	─	─	─	─	─	─	─	0
CT-AG-127	─	─	1	─	─	─	─	─	─	─	─	─	─	1
CT-AG-140	1	1	─	1	1	1	1	1	1	1	1	1	1	12
CT-AG-144	1	1	1	1	1	1	1	1	1	1	1	1	1	13
CT-AG-145	1	1	1	1	1	1	1	1	1	1	1	─	1	12
CT-AG-157	1	─	1	1	1	1	1	1	1	─	1	1	1	11
CT-AGAT-19	1	─	1	─	1	─	─	─	─	1	1	─	─	5
CT-AGAT-26	─	─	─	1	1	─	─	─	─	─	─	─	─	2
CT-AGAT-131	─	─	─	1	1	─	─	1	─	─	─	1	─	4
CT-CCA-71	─	─	1	1	─	─	─	─	1	─	1	─	─	4
CT-CCA-108	─	─	1	─	─	─	─	─	─	─	─	─	─	1
CT-CCA-137	1	1	─	1	1	─	1	1	1	1	1	1	─	10
No. of loci	14	11	17	17	16	12	12	15	17	15	17	15	11	

For loci that were successfully amplified, the number of alleles is given.

## Conclusions

For conservation purposes, 28 new microsatellite loci, including 12 monomorphic and 16 polymorphic loci, were isolated from *G. flabilabella*. The genetic diversity indices assessed using these 16 polymorphic microsatellite loci for the remained populations of this endemic and vulnerable species revealed that these markers are potentially useful for future studies, especially those focusing on evaluating the genetic variation and identifying distinct evolutionary units within populations for conservation management. Genetic diversity was characterized for three other related species using these 28 microsatellite markers. Furthermore, successful amplification in 13 other *Gastrodia* taxa indicated the transferability of these primer pairs. The interspecies transferability made these microsatellite loci useful for future research aiming to reconstruct the phylogeographic patterns and the process of speciation among closely related species. Additionally, the transferable microsatellite loci will be potentially useful for future studies that focus on establishing the standard operating system of molecular identification for *Gastrodia elata*, a traditional Chinese medicine.

## Abbreviations

Na: The number of alleles
Ne: The number of effective alleles
Ho: The observed heterozygosity
He: The expected heterozygosity
H: Shannon’s information index
F_IS_: The fixation index
H_WE_: The Hardy–Weinberg equilibrium
